# Short- and long-distance avian migrants differ in exercise endurance but not aerobic capacity

**DOI:** 10.1186/s40850-022-00134-9

**Published:** 2022-06-02

**Authors:** Steffen Hahn, Tamara Emmenegger, Sara Riello, Lorenzo Serra, Fernando Spina, William A. Buttemer, Silke Bauer

**Affiliations:** 1 Department Bird Migration, Swiss Ornithological Institute, Seerose 1, 6204 Sempach, CH Switzerland; 2 Molecular Ecology and Evolution Lab, Lund University, Lund, Sweden; 3 Riserva Naturale Statale “I sole di Ventotene e S.Stefano”, Ventotene, Italy; 4 Area Avifauna Migratrice, Istituto Superiore per la Protezione e la Ricerca Ambientale (ISPRA), Ozzano Emilia, Bologna, Italy; 5 School of Earth, Atmospheric, and Life Sciences, University of Wollongong, Wollongong, Australia

**Keywords:** Metabolic rate, Aerobic scope, Migration strategy, Passerine, Haemoglobin

## Abstract

**Background:**

Migratory birds differ markedly in their migration strategies, particularly those performing short- versus long-distance migrations. In preparation for migration, all birds undergo physiological and morphological modifications including enlargement of fat stores and pectoral muscles to fuel and power their flights, as well as cardiovascular and biochemical adjustments that improve lipid and oxygen delivery and uptake by flight muscles. While the magnitude of these changes varies in relation to migration strategy, the consequence of these variations on aerobic performance is unknown. We tested whether the aerobic performance of four Old-world flycatcher species (*Muscicapidae*) varied according to migration strategy by comparing minimum resting metabolic rates (RMR_min_), exercise-induced maximum metabolic rates (MMR), and exercise endurance times of short-distance and long-distance migratory birds.

**Results:**

As expected, RMR_min_ did not vary between short-distance and long-distance migrants but differed between the species within a migration strategy and between sexes. Unexpectedly, MMR did not vary with migration strategy, but MMR and blood haemoglobin content were positively related among the birds tested. Exercise endurance times differed substantially between migration strategies with long-distance migrants sustaining exercise for > 60% longer than short-distance migrants. Blood haemoglobin content had a significant positive effect on endurance among all birds examined.

**Conclusions:**

The lack of difference in RMR_min_ and MMR between long- and short-distance migrants during this stage of migration suggests that the attributes favouring the greater aerobic endurance of long-distance migrants did not come at the expense of increased maintenance costs or require greater aerobic capacity.

## Background

Migratory land birds are often characterized as being either short- or long-distance migrants based on the distances they cover, e.g. between higher-latitude breeding sites and lower-latitude non-breeding locations [1]. Although this distinction is more of a gradient than a sharp delineation, the typical short-distance migrant moves between breeding and nonbreeding sites within a continent or a biome, experiences predictable environmental conditions and finds plenty of landing opportunities along its route. In contrast, the typical long-distance migrant travels across continents and between biomes, with breeding and nonbreeding sites often separated by ecological barriers such as deserts, seas or mountain ranges [1]. Long-distance migrants face higher probability of experiencing unfavourable conditions during their journeys [2, 3], thus physiological and morphological adjustments promoting flight endurance should be favoured.

Short- and long-distance migrants typically undergo morphological and physiological changes in preparing for migration [4]. They increase lipid storage, pectoral muscle mass [5–7], heart mass [8, 9], flight muscle fibre volume [10], as well as increases in haematocrit and blood haemoglobin content [11, 12]. Along with these morphological modifications, several fatty acid binding proteins are upregulated that facilitate the transfer of fatty acids from the circulation system to myocytes as well as increases in key catabolic enzymes that promote the oxidation of mitochondrial fatty acids [13–17].

The increased proportion of metabolically active tissue such as the enlarged pectoral muscles and digestive organs in individuals during preparatory migratory stages result in elevated maintenance energy metabolism (measured as basal metabolic rate (BMR) or minimum resting metabolic rate (RMR_min_) [18, 19]. Furthermore, the enlarged pectoral and cardiac muscles in combination with the elevated blood hemoglobin concentration may are concomitantly related to increased maximum oxygen consumption rates [20, 21].

Although physiological adjustments are well-known in all migratory birds, it is unclear whether and in how far short- and long-distance migrants differ in aspects related to aerobic capacity. Earlier studies on migration-related physiological and biochemical adaptations have been mostly conducted in long-distance migrants, e.g. [14, 18, 22], which have revealed pronounced variation in relation to migration stage. However, studies of short- and long-distance migrating Palearctic passerines have also revealed striking morphological differences in birds at the same stage that differed in migration strategy. Histological examination of pectoral muscles showed long-distance migrants to have smaller fiber areas with higher capillary densities, resulting in shorter gas diffusion distances than muscles from short-distance migrants and resident birds [23]. As blood vessel supply and gas diffusion rate determine the rate of oxygen supply (and carbon dioxide removal) for flight muscles [24], diffusion distance directly affects the maximum achievable oxygen consumption rate and, potentially, the duration of aerobically powered activity of a bird.

To this end, we quantified three components of aerobic metabolic performance (RMR_min_; exercise-induced maximum metabolic rate, MMR; and exercise endurance) of four Old-world flycatcher species (*Muscicapidae, subfamilies Erithacinae and Saxicolinae*) that differ in migration strategy. The two short-distance migrants - European robins (*Erithacus rubecula*) and black redstarts (*Phoenicurus ochruros*) - spend their entire annual cycle within the Palaearctic and some populations move from North African non-breeding grounds to breeding sites in continental Europe [25]. Although tracking data are not available for these short-distance migrants, it can be assumed that suitable sites for stopping-over are not limited along the routes for these ubiquitous species. In contrast, the two long-distance migrants - common redstarts (*P. phoenicurus*) and whinchats (*Saxicola rubetra*) - overwinter in sub-Saharan Africa and migrate to Palaearctic breeding sites. During spring migration, they first cross the Sahara Desert where feeding is rarely possible, and some birds may shortly stage at sites around the Mediterranean Sea before resuming migration towards their breeding grounds [26–28]. Crossing the desert usually takes 2–3 days of extended flight bouts for a small passerine but non-stop flights also occur [29, 30].

We characterize strategy-dependent aerobic performances in actively migrating birds during their pre-breeding journey. We expected similar RMR_min_ across all species we studied irrespective of their migration strategy, because maintenance aerobic metabolism is generally determined by body size and body composition [31]. In contrast, we expect long-distance migrants to have higher aerobic capacity and longer exercise endurance times than short-distance migrants due to strategy-specific differences in flight muscle morphology and biochemical features [23].

## Results

### Body condition measures and haemoglobin content

**Fig. 1:**
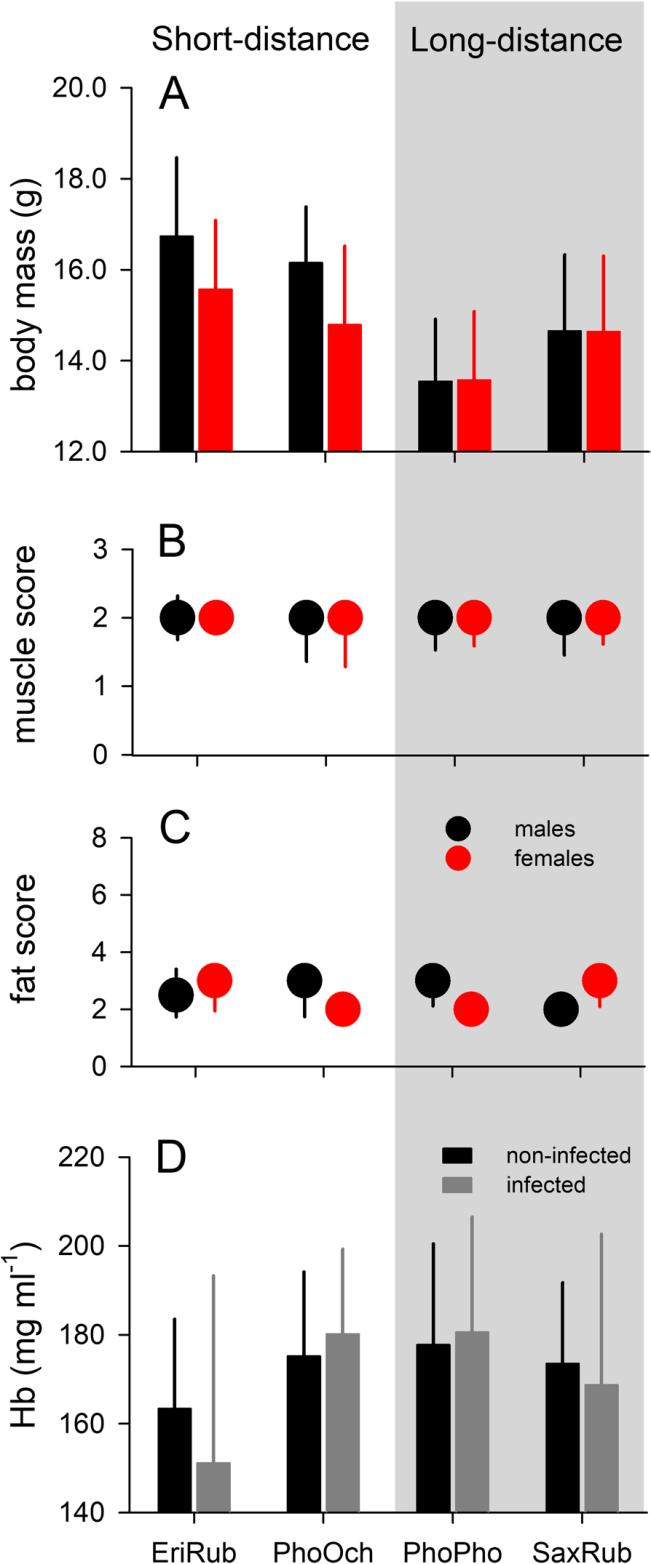
Body mass (in g, **A**), pectoral muscle scores (**B**), fat scores (**C**) as well as blood haemoglobin concentration (Hb, **D**, mg ml^− 1^) of European robins (EriRub) and black redstarts (PhoOch) as representatives for short-distance migrants as well as common redstart (PhoPho) and whinchats (SaxRub) as long-distance migrants. Body mass and Hb are given as means ± SD, muscle and fat scores are medians ±95% CI

Pectoral muscle scores varied between 1 and 2 and did not differ between short- and long-distance migrants, between infected and non-infected birds (*p* > 0.05) and differed marginally between sexes (*p* = 0.05, Fig. 1b, Table 1). However, within short-distance migrants, robins had higher average muscle scores than black redstarts (*p* = 0.02, Fig. 1b). Body fat scores were more variable, but migration strategy, sex or infection did not explain these variation (F = 1.15, *p* = 0.33, Fig. 1c, Table 1).

**Table 1:** Summary statistics of linear regression models testing for differences in body condition parameters

Factors	Pectoral muscle score (ranked)		Fat score (ranked)		Hb	
	t	p	t	p	t	p
Intercept	17.76	< 0.001	17.00	< 0.001	70.36	< 0.001
migration strategy	0.73	0.47	0.04	0.97	−1.89	0.06
w/i SD	2.44	0.02*	0.82	0.42	−2.85	0.004*
w/i LD	−0.29	0.77	0.25	0.81	1.78	0.08
Sex	1.95	0.05*	−0.05	0.96	0.18	0.85
Infection	−0.62	0.54	−2.03	0.04	−2.50	0.01*
F (df)	2.84 (5248)	0.02*	1.15 (5248)	0.33	5.18 (5244)	< 0.001*

Legend: Body condition estimates are pectoral muscle score, fat score, and haemoglobin concentration (Hb, mg ml^−1^). Hb refers to the haemoglobin content of whole blood. Migration strategy provides the comparison between long-distance (LD) and short-distance migration (SD), whereas w/i SD and w/i LD give the comparisons between species within each migration strategy. The factors sex and infection test for differences between males and females, and between non-infected and haemosporidian-infected birds. F, df, t and *p*-values are the accompanying statistical model parameters, * gives indicates statistically significant differences at *p* ≤ 0.05 (for details see Methods)

Average haemoglobin concentration ranged between 159.7 mg ml^− 1^ in robins and 167.5–175.9 mg ml^− 1^ in the other three species (Fig. 1d, Table 3). Haemoglobin concentrations did not differ between migration strategies or sexes but differed within short-distance migrants (Table 2). Birds infected with haemosporidian parasites had a lower haemoglobin concentration than non-infected birds (*p* = 0.01), especially in whinchats and robins (5 and 7% difference, respectively).

**Table 2:** Summary statistics of linear regression models testing for differences in aerobic capacity estimates

Aerobic capacity	Resting metabolic rate (RMR_min_)		Maximal metabolic rate (MMR)		Time to exhaustion	
	t	p	t	p	t	p
Intercept	−39.85	< 0.001	−14.95	< 0.001	−0.36	0.72
migration strategy	0.15	0.88	−0.14	0.88	−3.32	0.001*
w/i SD	4.27	< 0.001*	2.02	0.05*	−0.08	0.93
w/i LD	−3.26	0.001*	1.69	0.09	−1.50	0.14
sex	2.78	0.006*	−0.43	0.67	−2.16	0.03*
infection	0.01	0.99	2.47	0.01*	1.07	0.28
haemoglobin	−0.24	0.81	2.87	0.004*	3.07	0.002*
F (df)	6.54 (6176)	< 0.001*	2.97 (6240)	0.008*	7.15 (6241)	< 0.001*

Legend: Aerobic capacity estimates are the mass-specific resting and maximal metabolic rates (oxygen consumption rates, ml g^−1^ min^−1^), and the endurance exercise given as time to exhaustion (min). Migration strategy provides the comparison between long-distance (LD) and short-distance migration (SD), whereas w/i SD and w/i LD give the comparison between species within each migration strategy. Factors sex, infection, and the covariate haemoglobin test for differences between males and females, between non-infected and haemosporidian-infected birds as well as for the potential dependency with total haemoglobin in peripheral blood. F, df, t and p-values are the accompanying statistical model parameters, * indicates statistically significant differences at *p* ≤ 0.05 (for details see Methods)

### Aerobic performance

**Fig. 2:**
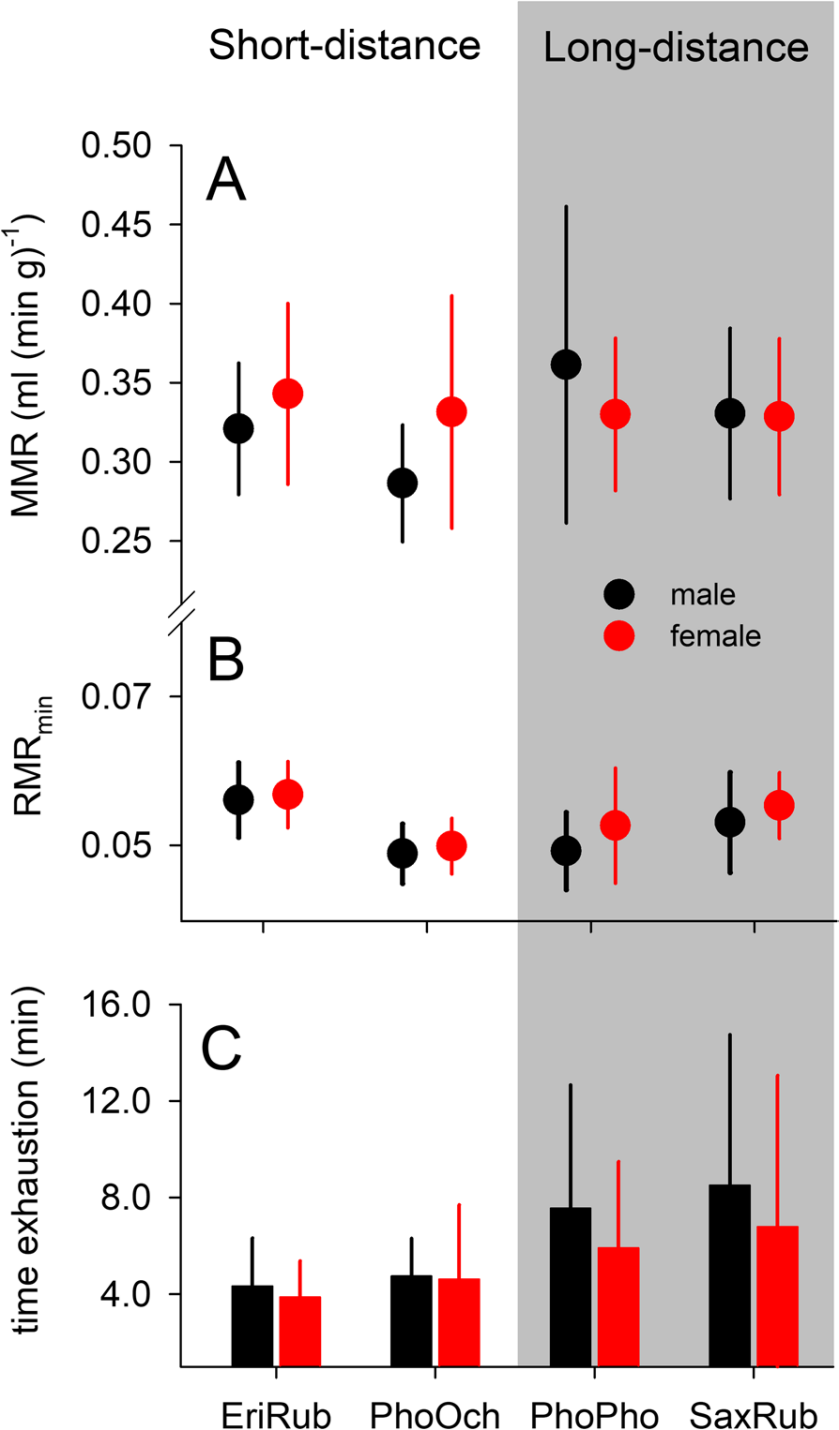
Aerobic performance of short-distance and long-distance migrants characterised by maximum metabolic rate (mass-specific MMR, V̇O_2_ ml min^− 1^, **A**), resting metabolic rate (mass-specific RMR_min_, **B**), time until exhaustion (min, **C**). European robins (EriRub) and black redstarts (PhoOch) are short-distance migrants as well as common redstart (PhoPho) and whinchats (SaxRub) are long-distance migrants. Black and red bars and dots indicate males and females, respectively. All data are means ± SD

RMR_min_ did not differ between short- and long-distance migrants but varied significantly within short-distance and within long-distance migrants (Table 2, Fig. 2b). Females had consistently igher median RMR_min_ than males, with differences of 3–6% depending on species (estimate: 0.02 ± 0.007; Fig. 2). Neither haemoglobin concentration nor blood parasite infection status was associated with RMR_min_ (Table 2). Considering body mass as a co-variate did not change these results.

Similarly, MMR did not differ between short- and long-distance migrants but differed slightly within short-distance migrants (Fig. 2). Sex did not influence MMR, but blood parasite infection had some effect (*p* = 0.01). In contrast, blood haemoglobin concentration was strongly associated with MMR (Table 2). Additionally, body mass significantly co-explained MMR, but the effect of other factors remained the same (for overview on species specific but unscaled aerobic capacity see Table 3).

**Table 3:** Descriptive statistics of species’ average body masses, oxygen consumption rates and haemoglobin concentration

Species	Migration strategy	Sex	Body mass (g)	RMR_min_ (ml min^− 1^)	N	MMR (ml min^− 1^)	n	Hb (mg ml^− 1^)	n
European robin *Erithacus rubecula*	SD	M	16.9 ± 1.88	0.862 ± 0.098	10	5.368 ± 0.879	14	154.4 ± 33	14
		F	15.2 ± 0.99	0.791 ± 0.076	19	5.340 ± 0.823	35	162.4 ± 19	35
Black redstart *Phoenicurus ochruros*	SD	M	16.5 ± 0.79	0.775 ± 0.070	7	4.623 ± 0.629	8	174.0 ± 23	8
		F	14.7 ± 1.78	0.689 ± 0.082	16	4.855 ± 0.906	22	174.5 ± 15	22
Common redstart *P. phoenicurus*	LD	M	13.6 ± 1.31	0.657 ± 0.088	39	4.737 ± 0.942	47	179.5 ± 16	48
		F	13.7 ± 1.33	0.692 ± 0.121	31	4.472 ± 0.770	44	171.6 ± 25	40
Whinchat *Saxicola rubetra*	LD	M	14.6 ± 1.70	0.742 ± 0.151	43	4.854 ± 0.895	56	166.6 ± 23	59
		F	14.7 ± 1.61	0.751 ± 0.084	21	4.813 ± 0.902	24	171.2 ± 19	24

Legend: Averages ± SD for male and female’ body masses (before MMR measurement), resting metabolic rates (RMR_min_), maximum metabolic rate (MMR) and haemoglobin concentration (Hb) of four Old world Flycatcher species differing in migration strategies (*SD* short-distance migrant, *LD* long-distance migrant). Data had been collected in birds during pre-nuptial spring migration

Time until exhaustion differed greatly between migration strategies with long-distance migrants sustaining exercise about 60% longer than short-distance migrants (Fig. 2c). Moreover, endurance times were statistically indistinguishable within the two short-distance migratory species and within the two long-distance migratory species (Table 2). Generally, females had 7–22% shorter endurance times than males. Finally, endurance times did not vary according haemosporidian infection status, but haemoglobin concentration showed a strong positive effect on endurance times (Table 2).

## Discussion

The extent of morphological and physiological adjustments in migratory birds is thought to be related to migration strategy [7, 23, 32]. In an earlier study, Lundgren and Kiessling [23] found differences in muscle composition between short-distance and long-distance migrants that likely have consequences for gas and metabolite exchange rates. Here, we examined the functional consequence of this by evaluating the aerobic capacity and exercise endurance of long-distance and short-distance migrating passerines during their migration.

As predicted, we found similar resting metabolic rates in short-distance and long-distance migrants. Contrary to our expectations, however, maximum metabolic rates did not vary between the two migration strategies. By contrast, exercise endurance times differed as we predicted, with long-distance migrants sustaining aerobic effort for much longer periods than short-distance birds under the same conditions. A plausible explanation for the similarities in aerobic metabolic rates is that short- and long-distance migrants of our study system had similar physiological and morphological constitution associated with RMR and MMR at this stage of migration, and endurance time is not primarily determined by aerobic capacity, at least not in individuals in good body condition.

The body composition of migratory passerines can vary considerably over the course of migration. For example, the body mass of migratory garden warblers (*Sylvia borin)* reaches its highest level before flying across the Sahara [32] with most of this due to fat accumulation and enlargement of pectoral muscles. After initial departure, body fat, but also flight and heart muscles had declined by 30, 25 and 23%, respectively [33]. Such variations of body constituents are probably accompanied by changes in metabolic rates [20, 22, 34, 35]. Irrespective of migration strategy, similar-sized birds undertaking the nocturnal endurance flight, here across the Tyrrhenian Sea, face the same energy demands to reach the goal. This constrain is equally for short and long-distance migrants, making it likely that their body composition and constitution is similar.

This interpretation is supported by field observations, showing that average fat scores in robins and black redstarts, the short-distance migrants, were similar to scores of common redstarts and whinchat, the long-distance migrants, although the latter showed higher variability [36, 37]. Our selection of individuals with fat scores > 0 (see methods) levelled some of the variation in body condition across species and thereby allowed testing of migration strategy net effects under comparable circumstances.

### Variation in resting metabolic rate

Resting/basal metabolism in birds reflects the overall energy required for maintenance of metabolically active tissues and organs, which generally varies with body mass, body organ proportions, phylogeny, and season [19, 20, 38, 39]. Maintenance energy metabolism, in our case RMR_min_, should not be constrained by the circulatory system and gas exchange to muscle cells as these are dimensioned for higher, maximal workloads [24, 40]. Consequently, migrants of similar size and body organ proportions, from similar zoogeographic regions and measured during the same periods of their annual cycle should show similar maintenance metabolic rates independent of migration strategy – an expectation that our results support for the four passerine species (Fig. 2b).

Apart from similar mass-specific RMR_min_ across our study species (and migration strategies), we note two other interesting patterns. First, the RMR_min_ values we measured in these migrating passerines averaged 11.7% higher than predicted from allometric analysis of basal metabolic rates (BMR) for north-temperate passerines [38]. As BMR measurements explicitly exclude all life stages associated with elevated energy requirements such as growth, moult, reproduction, and migration, it is unsurprising that resting metabolic rates were higher than predicted BMR. As a phenotypically flexible trait [39], RMR_min_ is known to rise during pre-migration and migration periods in passerine birds [21], likely reflecting the energetic costs needed to prepare and maintain migratory capability. Second, the mass-specific RMR_min_ differed between sexes, with females having on average a 3.9% higher RMR_min_ than males (Fig. 2b). These differences are intriguing, particularly as there were no obvious differences in examined body morphology (Fig. 1b and c). However, one aspect that may account for this difference, and that is shared by long-distance and short-distance migrants, is the transition from the migration life-history to breeding life-history stages, wherein gonadal development is initiated [33, 41]. Unfortunately, the metabolic costs of sexual maturation are not well known, with available evidence showing a 12% increase in RMR_min_ in females during pre-laying compared with non-breeding periods [42]. Whether the increased RMR_min_ in females of our long-distance and short-distance migrants relates to a protogynic gonadal development requires further study (see also [43]).

### Variation in maximal metabolic rates

Contrary to our expectations, we found no effect of migration strategy on MMR, indicating that short- and long-distance migrants had similar aerobic capacity during this phase of migration.

We also found species-specific MMRs (Table 3) averaged 9% higher than expected from allometric analysis [39]. This discrepancy might result from biased sampling of species underlying this predictive equation, which was mainly based on tropical passerines with lower BMR and MMR than those of temperate birds [44]. However, MMR has been shown to vary with life-history stage, with increases occurring in preparation for and during migration periods [21].

The aerobic capacity can be constrained if sufficient oxygen is not supplied to the muscles. The increased locomotor demands of sustained flight needs commensurate improvements in blood oxygen carrying capacity, which is usually attained through seasonally increased blood haemoglobin concentration during migration [12, 45]. Improved oxygen supply also depends on increased rates of gas exchange between the blood and myocytes, which is facilitated by a greater extent of vascularisation in the muscle [23]. We found a generally high blood Hb in all migrants, irrespective of migration strategy (Fig. 1d). In most cases, the median Hb contents of the four species exceeded those measured in some trans-Saharan passerine migrants at the early stages of their autumnal desert crossing [46]. Moreover, we verified that, independent of migration strategy, birds with high Hb also showed high MMRs. Thus, MMR and haemoglobin concentration seem to be mechanistically linked in short- and long-distance migrants.

That short- and long-distance migrants had similar RMR_min_ and MMR but differed in exercise endurance times (see below) implies that factors other than aerobic capacity are involved. An alternative explanation might be that that our MMR measurements did not reflect the birds’ upper aerobic limit, despite all birds being exhausted at the conclusion of exercise. However, we believe our MMR measurements are representative of aerobic capacity for several reasons. First, other studies have found MMR measurements in hop-flutter wheels were highly repeatable over time, implying that all birds tested reach consistent aerobic limits [47, 48]. Second, the plasma metabolite profiles of long-distance migrating Red-eyed vireos (*Vireo olivaceous olivaceous*) undergoing MMR measurements revealed that they were fuelling this activity with the same lipids as wild passerines during nocturnal migratory flights [49]. Finally, all birds were exhausted when we terminated their MMR measurements suggesting that both short and long-distance migrants in our study had reached their aerobic limits.

### Variation in exercise endurance

Our most striking result was that long-distance migrants had significantly greater exercise endurance than short-distance migrants at the same level of aerobic exertion during maximal exercise. Long-distance migrant species have greater pectoral muscle enzyme activities involved with fat catabolism than short-distance species, rendering them capable of relying on lipid reserves to satisfy metabolic needs over a broader range of flight demands [13, 14, 17, 50].

The transfer of plasma-borne metabolic substrates and oxygen to flight muscle myocytes and the subsequent removal of metabolic products relies on diffusion, with distance between exchanging surfaces having profound effects on exchange rates [51]. Thus, birds differing in aerobic energy demands are likely to also differ in vascular characteristics. Indeed, birds with different migratory strategies substantially differed in muscle fibre diameters and blood capillary density per unit of muscle fibre area [23] with the latter being highest in long-distance migrants, intermediate in short-distance and lowest in partial/non-migratory populations. Seasonal variation in muscle fibre diameter has recently been found in snow buntings (*Plectrophenax nivalis*) switching from residence to migratory phases, which increases muscle power output while lowering energy costs of muscle maintenance [10]. Such morphological adjustments are expected to affect rates of fuel and oxygen provision to flight muscles as well as metabolic end-product removal, and consequently, on aerobic endurance. The positive relation between Hb and endurance time in our study supports this view. To our best knowledge, Lundgren and Kiessling [23] is the only published study investigating muscle fibre type and vascularisation in 16 Palaearctic passerine species of various migration strategies. However, the study included only one of our study species (the European robin), and we do not know if the other species in our study show the same histological composition.

Besides these anatomical adaptations, there is also the possibility that the long-distance and short-distance migrating species differ in their mitochondrial characteristics. For instance, [52] showed that mitochondrial respiration in migratory and sedentary subspecies of Yellow-rumped warblers (*Setophaga coronata* ssp.) differs with higher efficiency of ATP production per unit of oxygen consumed in the migratory subspecies. If long-distance and short-distance migrants differ in their ATP production, we would expect them to also differ in the expression of genes encoding mitochondrial OX-PHOS pathways, although this remains speculative at this point.

## Conclusions

We conclude that the higher exercise endurance of long-distance compared to short-distance Palaearctic-African passerine migrants cannot be explained by traditional measures of body condition (fat and pectoral muscle scores) or measures of RMR_min_ and MMR. Because the aerobic expenditure during exercise measurements did not differ between migration strategies, a functional explanation accounting for differences in aerobic endurance awaits additional physiological and biochemical appraisal as well as thorough parallel genetic and morphological analyses.

## Methods

Adult birds were captured by mist-netting during their spring migration between March and May in 2016 and 2017 on Ventotene island, central Mediterranean Sea (40.79°N, 13.42°E). Ventotene is the first landing opportunity for passerine migrants that fly across the Tyrrhenian Sea from North-Africa (Tunisia, distance 480 km) or from Sicily (distance 300 km) to the central part of the Apennine peninsula [37]. Birds were ringed and sexed according to morphological or subsequent genetic characterisation (robins only, see below). Additionally, we determined the presence of haemosporidian infections among individuals, which may compromise aerobic performance [21, 53] (see Table 4 for sample size).

**Table 4:** Study species, their migration strategy and sample sizes

Species	Migration strategy	RMR_min_	MMR/ Time exhaustion/Hb	Haemosporidian prevalence (%)
European robin	SD	30 (10 /19 /1)	50 (14 /35 /1)	34.0
Black redstart	SD	23 (7/16/0)	30 (8/22/0)	33.3
Common redstart	LD	70 (39/31/0)	91 (47/44/0)	37.0
Whinchat	LD	64 (43/21/0)	83 (56/24/3)	68.7

Legend: Migration strategy refer to short-distance (SD) and long-distance (LD) migration. Sample sizes are given as the number of individuals for measurements of minimum resting (RMR_min_) as well as maximum metabolic rate (MMR) including the time to exhaustion and haemoglobin concentration (Hb) and the species-specific prevalence of haemosporidian infection. The numbers refer to total n, and n males/ n females, and n unknown sex (in brackets). RMR_min_ was determined in a randomly selected subsample

### Body condition, haemoglobin, and haemosporidian infections

We measured body mass (to the nearest 0.1 g), and assessed body condition at capture by scoring body fat stores (scores from 0 to 8) and the size of the pectoral muscle (scores from 0 to 3) based on EURING standard protocols (sensu [54]). These scores are unaffected by differences in structural body size and, thus, provide valid estimates of relative body condition among similar species [55]. We excluded birds with fat scores < 1 from our metabolic analyses to avoid bias due to starvation-induced alteration of their physiology [56]. All morphometric measures were taken as part of the long-term ringing programme of the Istituto Superiore per la Protezione e la Ricerca Ambientale (ISPRA), Ozzano dell’Emilia, Italy. Birds had not been anesthetized neither during ringing nor during oxygen consumption measurements.

After each MMR measurement (see below), we collected about 35 μl of blood into heparinised capillary tubes following puncture of a brachial vein to determine total blood haemoglobin concentration (Hb, mg ml^− 1^) using a HemoCue Hb201 and to make blood smears. The remaining blood was stored in SET buffer for haemosporidian detection and genetic sexing (robins only).

Endoparasites can affect the metabolic rates of birds [53, 57]. Thus, we checked all individuals for blood parasite infection (*Haemoproteus* and *Plasmodium spec)* using both molecular analysis and microscopy of blood smears. We screened DNA extracts from whole blood for parasite DNA by qualitative PCRs (for details on procedure see [58]. About one to two third of individuals per species were infected with haemosporidians (for species-specific prevalence see Table 4). We determined the intensity of blood parasite infection by light microscopy of Giemsa-stained blood smears (see [58]. Median parasitaemia of infected birds was generally low (< 0.1‰), which is typical for chronic infections [59]. We included haemosporidian infection status as a binary factor in the statistical analysis (see statistical analysis).

### Aerobic performance

We characterised aerobic performance by measuring an individual’s maximal metabolic rate (MMR) and minimum resting metabolic rate (RMR_min_) using flow-through respirometry. We also determined endurance, i.e., the time from the onset of MMR activities until birds reached exhaustion (time until exhaustion, min) during enforced exercise.

For endothermic animals, RMR_min_ is the minimum oxygen consumption (minV̇O_2,_ ml min^− 1^) rate when they are post-absorptive, asleep during the rest-phase of their daily cycle and exposed to thermoneutral temperatures. RMR_min_ represents the maintenance energy requirements of an animal at its concurrent life history stage and is equivalent to basal metabolic rate if the individual is non-growing and non-reproductive.

MMR is the maximum rate of oxygen consumption (maxV̇O_2,_ ml min^− 1^) during enforced exercise. In wild birds, this is best achieved using a hop-flutter wheel, with rotation speed dynamically adjusted to encourage repeated take-offs [20, 39]. We measured MMR of birds shortly after capture and therefore, consider these measurements a proxy for maximal achievable O_2_ consumption rates after sea crossing. The MMR system had an effective volume of 9.25 l and was supplied with dry compressed ambient air at a rate of 5 l min^− 1^ using a calibrated mass-flow controller (MKS Instruments). The rotation speed of the hop-flutter wheel was manually adjusted to each bird’s behaviour and fully stopped when the bird could not hold its position in the wheel (which was recorded as the time of exhaustion). Oxygen consumption during exercise was continuously recorded with an FC1 oxygen analyser (Sable Systems, NV, USA), using inlet air as a reference at the start and end of each measurement period. Sampled air was drawn through a tube containing Drierite and soda lime to remove water vapour and CO_2_, respectively, before reaching the oxygen analyser. MMR was computed from the highest instantaneous oxygen consumption rates measured over a 30s interval of exercise, after baseline correction and smoothing the data to remove electrical noise (1 s smoothing interval over three cycles). All data were processed using LabHelper and LabAnalyst software (http://warthog.ucr.edu), we used the instantaneous correction option to finally determine instantaneous oxygen consumption rates [60].

Due to logistical constraints, we measured RMR_min_ in a randomly selected subsample of birds used for MMR (see Table 4 for sample sizes). Following MMR measurements, randomly selected birds were placed in holding cages under natural lighting and had free access to water and food (a mixture of live mealworms (*Tenebrio* spp.) and dry food (Insect pate, Orlux Versele-Laga, BE). The food was removed from cages three hours before birds were placed in 4.0 l respirometer chambers, each fitted with a perch and provided compressed atmospheric air dried through a silica gel column and maintained at 500 ml min^− 1^ by mass flow controllers (Tylan Model FC-280S). The respirometers were housed in a cabinet maintained at 30 °C, which is thermoneutral for birds of this size [61]. Oxygen consumption rates (V̇O_2_) were evaluated by comparing oxygen content of inlet and outlet air for each chamber after removal of water vapour and CO_2_ by passing air through Drierite and soda lime, respectively, before being directed to Oxzilla II Differential Oxygen Analyzers (Sable Systems, NV, USA). Voltage outputs from the oxygen analysers were recorded at 5 s intervals and each bird was sampled continuously for 27 min per hour between 19:00 h and 06:00 h, with RMR_min_ determined from the lowest 5-min running average of VO_2_ measured during the final 6 h of the experiment [60]. Our multiplexed system allowed to measure RMR_min_ of eight birds per night by using two oxygen analysers in parallel. Finally, we measured body mass of birds (to nearest 0.1 g) after MMR, and before and after birds undertook RMR_min_ measurements. All birds were released on completion of these evaluations.

### Statistical analysis

First, we checked for species and sex-specific body mass differences using analysis of variance (ANOVA). There were significant and consistent mass differences between the study species and between sexes in the RMR_min_ and the MMR data set (RMR_min_ data set: F_4,112_ = 8.15, *p* < 0.001, MMR dataset: F_4,248_ = 19.84, *p* < 0.001; Fig. 1a). The largest average difference of 15% occurred between European robins and common redstarts (Fig. 1a), and females were lighter than males in two of four species (see Table 3 for detailed data). As body mass is the main predictor for metabolic rates in general but we were mainly interested in their relative variation dependent on migration strategy, we scaled and log-transformed all RMR_min_ and MMR measures by body mass for subsequent analysis. Finally, we analysed how metabolic and body condition measures related to migration strategy and species by constructing a set of linear mixed effect models using R packages lme4 [62], and arm [63]. For these models, we included migration strategy and species by defining a contrast matrix, which allowed us testing for differences a) between short- and long-distance migrants, b) within short-distance migrants, and c) within long-distance migrants. Please note that defining a contrast matrix, i.e. recoding (categorical) variables into a set of “contrast variables” may seem unusual but is used to avoid including both ‘species’ and ‘strategy’ and thereby over-specifying the model. A contrast matrix only arranges the differences between species such that, e.g., one model parameter is defined as the difference between long- and short-distance migrants.

We also included additional covariates in the linear models namely fat and muscle score estimates, sex, infection status, haemoglobin concentration and body mass to test for their influence on metabolic rates (Table 5). Statistics had been run in R3.6.1; for the availability of R-codes see below.

**Table 5:** Overview about the structure of linear mixed effect models

Target variable	Co-variates
Fat score ~	Mig-strategy/species + sex + infection
Muscle score ~	Mig-strategy/species + sex + infection
Haemoglobin Hb ~	Mig-strategy/species + sex + infection
log10(mass-specific RMR_min_) ~	Mig-strategy/species + sex + infection + Hb
	Mig-strategy/species + sex + infection + Hb + log Body mass
log10(mass-specific MMR) ~	Mig-strategy/species + sex + infection + Hb
	Mig-strategy/species + sex + infection + Hb + log Body mass
T_Exhaust_ ~	Mig-strategy/species + sex + infection + Hb

## Data Availability

R-codes of all analyses are available on GitHub (https://github.com/silkebauer/MigStrategy_Aero) and will be made permanently available on Zenodo (see below). The dataset supporting the conclusions of this article is available on Zenodo (https://zenodo.org/communities/vora/about/) under 10.5281/zenodo.6521939(V2).
